# Leveling and abuse among patients with bipolar disorder at psychiatric outpatient departments in Ethiopia

**DOI:** 10.1186/s12991-017-0152-4

**Published:** 2017-07-11

**Authors:** Habte Belete

**Affiliations:** Psychiatry Department, College of Medicine and Health Science, Bahir Dar University, P.O. Box: 79, Bahir Dar, Ethiopia

**Keywords:** Abuse, Physical, Verbal, Bipolar, Ethiopia

## Abstract

**Introduction:**

The World Health Organization (WHO) clearly states the importance of psychological well-being in the definition of health as “a state of complete physical, mental and social well-being and not merely the absence of disease or infirmity”. However, in the community, the lives of people with bipolar disorders are often harsh and abusive. Till now, the rate and related information concerning verbal or physical abuse among patients with bipolar disorder at psychiatric outpatient clinics have not been well addressed in Ethiopian settings.

**Methods:**

Data were collected by interviewing 411 systematically selected participants at outpatient department of Amanuel Mental Specialized Hospital. For analysis, logistic regression and adjusted odds ratios (AOR) with 95% confidence intervals (CI) were used, and *P*  < 0.05 was considered statistically significant.

**Results:**

The prevalence of abuse (verbal/physical) was 37.7%. Having two or more episodes [AOR 1.70, 95% CI (1.06, 2.74)], a history of aggression [AOR 3.06, 95% CI (1.63, 5.75)] and comorbid illness [AOR 2.21, 95% CI (1.25, 3.90)] were significantly associated.

**Conclusion:**

The prevalence of reported abuse is high among patients with bipolar disorder, and it is important to remember the rights of patients during treatment.

## Introduction

Bipolar disorder is a chronic and severe mental disorder that affects the adult population worldwide. Bipolar disorder causes substantial psychosocial morbidity that affects the patient’s marriage, social contacts, occupation, communication and other aspects of life. Even if the behavior of patients with bipolar disorder is somewhat challenging to caregivers [1–3], the responses of families or caregivers to this pathological behavior should not be inhuman. The challenge to caregivers or families is not only the abnormal behavior, but also the cost of their treatment. Individuals with bipolar disorder have high rates of psychiatric and medical comorbidity, which contribute to increased utilization of healthcare resources [4]. WHO clearly states the importance of psychological well-being in the definition of health as “a state of complete physical, mental and social well-being and not merely the absence of disease or infirmity” [5]. However, in the community the lives of people with bipolar disorders are often harsh and abusive [6]. The rate of mood disorder occurrence reaches up to 52% even among healthy individuals who have suffered from abuse or violence in their lifetimes [7]. Patients with bipolar disorder attempt suicide at a rate of at least 25 to 50% [8], and most of them complete their death wish. Psychosocial factors such as abuse have an association with suicidal attempts [9]. The problem of reported abuse (physical and verbal) in individuals with severe mental illness has received relatively little attention, even though several studies suggest an extremely high prevalence of victimization in this population.

There is a high prevalence of victimization by abuse (mainly physical abuse) among patients with bipolar disorders and other severe mental illnesses. Studies have revealed that physical or emotional abuse can take place because of the presence of psychosocial factors, demographic factors and living circumstances, and substance abuse [10–12].

Many studies find that patients with bipolar disorder, especially women, suffer a high rate of physical and emotional abuse [13, 14], as do homeless patients with episodically severe mental illness [15].

Unfavorable childhood experiences and stressful life events significantly predict recurrence [16]. Unfavorable experiences contribute to worsening mental and physical health and lead to poor outcomes among adults with severe mood disorders [17]. Most patients with bipolar disorder who had unfavorable life events such as physical abuse are prone to rapid cycling, suicidal behavior, early onset and higher comorbidity for other psychiatric and cognitive disorders [18, 19].

Severe emotional abuse among patients with bipolar disorder is associated with lifetime substance misuse comorbidity and rapid cycling. A history of childhood abuse among patients with bipolar disorder has an association with lifetime suicidal attempts. Multiple forms of abuse show a graded increase in the risk of suicidal attempts, rapid cycling and poor prognosis among patients with bipolar disorder [20]. Patients' exposure to abuse at an early age is associated with poor response to treatment, risk of hospitalization and an increase in residential treatments [21]. A history of abuse is common among patients with bipolar disorders [22–24]. Current reported abuse and psychosocial stressors lead to worsening morbidity [25] and increases mortality from suicide. Stressful life events carry a risk of suicidal behaviors among patients with bipolar disorders [26, 27]. In general, bipolar disorder affects the patient and families. Therefore, describing the challenge to patients and social systems is important to improve health care and to provide good mental health care in low income countries.

The prevalence of physical abuse among patients with psychiatric illnesses, including bipolar disorder, is reported to be high in studies from western countries. For instance, an American study stated that 62.8% of the participants reported physical abuse from their partners, and 45.8% of the participants reported abuse from their own family members [28]. The prevalence of physical abuse was 27.3% among patients with bipolar disorder and schizophrenia. The prevalence of adulthood physical abuse was higher among patients with major psychiatric disorders (bipolar disorders and schizophrenia) compared to patients with medical illnesses [29]. Among patients with mood disorders, the reported abuse was 35%, including various types of abuse, but 75% reported physical abuse [30].

While the western literature describes the issue of abuse among patients with bipolar disorder well, there are still few data concerning abuse among patients with bipolar disorder in Ethiopia. The nationwide prevalence of bipolar disorder is not well reported in Ethiopia. Mental health services are quiet poor, and there is only one mental hospital (Amanuel Mental Specialized Hospital) for the country. Most rural communities choose cultural, traditional and religious healing methods in Ethiopia. However, many cultural treatments for mental illness are prone to physical or verbal abuse such as insults and restraints. Therefore, the aim of this study was to determine the magnitude of abuse among patients with bipolar disorder at psychiatric outpatient clinics in Ethiopia.

## Methods

### Study setting

A cross-sectional survey was conducted at the outpatient department of Amanuel Mental Specialized Hospital in Addis Ababa, Ethiopia. It has 15 outpatient departments; 8 of them serve approximately 11,500 bipolar patients yearly. The hospital has 300 beds for adult psychiatry inpatients and emergency, forensic and addiction services. While there are psychiatric clinics in many different general and specialized hospitals in Ethiopia, Amanuel Mental Specialized Hospital is the only mental hospital in Ethiopia that hosts many referral cases from throughout the country. The number of follow-up patients with bipolar disorder in this hospital seems unusual. However, the hospital is the only mental hospital in Ethiopia that hosts many patients with relapse and chronic illness from all parts of the country.

### Participants

The source population was participants who had been clinically diagnosed (according to the Diagnostic and Statistical Manual of Mental Disorders, 5th edition) with bipolar disorder (any type of bipolar disorder) by psychiatrists and mental health professional specialists (who have master's degrees in mental health). The patients had bipolar disorder and had been regularly followed up at Amanuel Mental Specialized Hospital, which sees a yearly average of 11,500 patients. The number of patients was estimated by taking the annual average patient follow-up by year at Amanuel Mental Specialized Hospital. The hospital uses the Diagnostic and Statistical Manual of Mental Disorders, 5th edition, to diagnose mental disorders including bipolar disorder. Those participants who presented to the outpatient departments during the study period and met the inclusion criteria comprised the study population. The study participants were aged 18 years and above; those who were seriously ill (emotionally disturbed and unable to maintain normal conversation) and incapable of communication were excluded.

From the total 423 potential participants, 411 completed the interview, but 5 declined, 4 were unable to complete the form, and 3 were excluded for not meeting the criteria. A systematic random sampling technique was used to select participants by using the sampling fraction of 2 of 958 patients with bipolar disorder (who visit the hospital each month on average). Data were collected by well-trained, degree-holding psychiatric nurses by interviewing patients using a questionnaire translated into the local language, Amharic, which is the national working language of Ethiopia. The Ethical Review Board of the University of Gondar and Amanuel Mental Specialized Hospital approved the study, and written consent was taken from the participants. Confidentiality was maintained throughout the process by anonymous questionnaires and omitting personal identification.

### Instrument

The reported physical or verbal abuse was assessed by questioning the patients and relatives and reviewing their medical records for whether or not the patients had suffered abuse (physically/verbally) from their caregivers or family members since their morbidity. The questionnaire was developed for this study. It considers physical abuse to have occurred if any of the following happened to the patients: hitting, slapping, pushing or throwing any material to harm the patient. For verbal abuse, any of the following were considered: shouting, insulting, criticizing and undermining the patient’s role in the family, also by the caregiver, after the patient had been diagnosed with bipolar disorder. This tool had internal consistency (Cronbach’s alpha) of 76% and was understood during the pretest. Socioeconomic status (wealth) of the study participants was assessed by using principal component analysis in which eigenvalues greater than 1 were used as extractions, and the factors to extract were fixed at five (from the lowest to highest). Participants’ educational status was classified as educated or not based on whether they were receiving formal education.

Medication adherence was assessed by a standard tool, the Morisky Medication Adherence Scale 8-item tool, and levels were set as poor adherence (0–6), moderate (≥6 and <8) and good (8) on the 8-item Morsiky Medication Adherence Scale [31]. Social support was assessed using the Oslo-3 Social Support Scale, with categories of strong (12–14), moderate (9–11) and poor social support (3–8) [32]. Perceived stress was assessed by the Global Perceived Stress Scale 10-item tool, and its health concern stress level was categorized into scores of 0–11 (low), 12–15 (moderate) and 16 and above (high) [33]. Current substance use (smoking, alcohol and khat) was assessed by adopting the alcohol, smoking and substance involvement screening test [34]. Other clinical variables such as duration of the illness, age of illness onset and history of aggression were assessed by reviewing the patients’ chart and self-report.

### Analysis

Data were entered into Epi Data version 3.1 and analyzed using the Statistical Package for Social Sciences, version 20. Descriptive statistics were analyzed, and multivariate and binary logistic regression analysis was used to select the predictors associated with abuse. Association was determined using odds ratios by taking 95% confidence intervals, and *P* < 0.05 was considered statistically significant.

## Results

The study included a total of 411 respondents with bipolar disorder, and 236 (57.4%) were females. The mean age of participant was 34.35 years (Standard Deviation: 34.35 ± 11.13 years), and most of the participants 142 (35.4%) were of Amharic ethnicity (Table 1).

**Table 1:** Demographic predictors related to abuse in bipolar patients

Characteristics	Abuse		Overall	Chi-square (*X* ^2^) (*P* value)
	Yes	No		
Sex				
Male	71 (45.8%)	104 (40.6%)	175 (42.6%)	1.06 (0.30)
Female	84 (54.2%)	152 (59.4%)	236 (57.4%)	
Residency				
Urban	117 (75.5%)	177 (69.1%)	294 (71.5%)	1.91 (0.17)
Rural	38 (24.5%)	79 (30.9%)	117 (28.5%)	
Education				
No formal education	17 (11%)	52 (20.3%)	69 (16.8%)	6.04 (0.01)
Educated	138 (89%)	204 (79.7%)	342 (83.2%)	
Religion				
Christian	115 (74.2%)	196 (76.6%)	311 (75.7%)	0.29 (0.56)
Muslim	40 (25.8%)	60 (23.4%)	100 (24.3%)	
Marital status				
Currently married	58 (37.4%)	122 (47.7%)	180 (43.8%)	4.11 (0.04)
Not married	97 (62.6%)	134 (52.3%)	231 (56.2%)	
Job				
Has job	111 (71.6%)	188 (73.4%)	299 (72.7%)	0.16 (0.45)
No job	44 (28.4%)	68 (26.6%)	112 (27.3%)	
Ethnicity				
Amahara	60 (38.7%)	82 (32%)	142 (34.55%)	3.6 (0.46)
Oromo	48 (31%)	87 (34%)	135 (32.85%)	
Gurage	32 (20.6%)	50 (19.5%)	82 (20%)	
Tigray	6 (3.9%)	18 (7%)	24 (5.8%)	
Others^a^	9 (5.8%)	19 (7.5%)	28 (6.8%)	
Wealth				
Lowest	35 (22.6%)	48 (18.8%)	83 (20.2%)	2.33 (0.67)
Second	34 (21.9%)	54 (21.1%)	88 (21.4%)	
Middle	27 (17.4%)	51 (19.9%)	78 (19%)	
Fourth	35 (22.6%)	52 (20.3%)	87 (21.2%)	
Highest	24 (15.5%)	51 (19.9%)	75 (18.2%)	

^a^Wolaita, Sidama, Gamo

Of the total participants, 139 (33.8%), 84 (20.4%) and 69 (16.8%) had anxiety symptoms, guilty feelings and grandiosity during the study period, respectively. Among total participants, 52.8% had >5 years morbidity (Table 2).

**Table 2:** Psychosocial and clinical factors related to abuse among patients with bipolar disorder

Characteristics	Abuse		Overall	Chi-square (*X* ^2^) (*P* value)
	Yes	No		
Duration of illness				
Less than a year	5 (3.2%)	15 (5.9%)	20 (4.9%)	5.06 (0.06)
1–5 years	57 (36.8%)	117 (45.7%)	174 (42.3%)	
>5 years	93 (60%)	124 (48.4%)	217 (52.8%)	
Age at onset of illness				
Before 18 years old	30 (19.4%)	38 (14.8%)	68 (16.5%)	6.74 (0.034)
Between 18 and 24 years	69 (44.5%)	92 (36%)	161 (39.2%)	
After 24 years	56 (36.1%)	126 (49.2%)	182 (44.3%)	
History of aggression				
Yes	141 (91%)	191 (74.6%)	332 (80.8%)	16.64 (0.00005)
No	14 (9%)	65 (25.4%)	79 (19.2%)	
Medication adherence				
Poor	51 (32.9%)	66 (25.8%)	117 (28.4%)	2.75 (0.25)
Moderate	57 (36.8%)	111 (43.4%)	168 (40.9%)	
Good	47 (30.3%)	79 (30.8%)	126(30.7%)	
Perceived stress				
Low	43 (27.7%)	75 (29.3%)	118 (28.7%)	0.48 (0.78)
Moderate	34 (22%)	49 (19.1%)	83 (20.2%)	
High	78 (50.3%)	132 (51.6%)	210 (51.1%)	
Comorbid illness				
Yes	35 (22.6%)	27 (10.5%)	62 (15.1%)	10.91 (0.001)
No	120 (77.4%)	229 (89.5%)	349 (84.9%)	
Social support				
Poor	63 (40.6%)	79 (30.9%)	142 (34.5%)	4.15 (0.13)
Moderate	62 (40%)	122 (47.6%)	184 (44.8%)	
Strong	30 (19.4%)	55 (21.5%)	85 (20.7%)	
Khat chewing				
Yes	33 (21.3%)	34 (13.3%)	67 (16.3%)	4.54 (0.03)
No	122 (78.7%)	222 (86.7%)	344 (83.7%)	
Tobacco use				
Yes	38 (24.5%)	39 (15.2%)	77 (18.7%)	5.46 (0.02)
No	117 (75.5%)	217 (84.8%)	334 (81.3%)	
Alcohol use				
Yes	52 (33.5%)	61 (23.8%)	113 (27.5%)	4.58 (0.03)
No	103 (66.5%)	195 (76.2%)	298 (72.5%)	

### Magnitude of abuse

The magnitude of reported abuse (physical or verbal) was 37.7%. This happened after patients’ diagnosis with bipolar disorder. Concerning the type of abuse, of the total participants, 104 (25.3%) reported physical abuse. For verbal abuse, 134 (32.6%) reported insults, 129 (31.4%) were criticized, 42 (10.2%) were sent away at mealtime, 138 (33.6%) were shouted at by their family members, and 53 (12.9%) were restricted from their role in the family. All patients who reported abuse also reported verbal abuses at least once during their morbidity.

Of the total patients who reported abuse, 84 (54.2%) were females; 121 (78.1%) had more than one episode, and 63 (40.6%) had poor social support. Patients who reported abuse perceived that their life was full of stress; 78 (50.3%) were scored as having high perceived stress, and 123 (79.4%) used some of these substances (alcohol, tobacco and khat).

### Multivariate analysis

After multivariate analysis of abuse in relation to all explanatory variables, a history of aggressive behavior, having two or more episodes of bipolar disorder and having a comorbid illness were found to be more statistically significant than other variables (Table 3).

**Table 3:** Factors related to abuse in bipolar patients

Explanatory variables	Abuse (physical/verbal)		(Crude odds ratio) (95% CI)	Adjusted odds ratio (AOR) 95% CI
	Yes	No		
Marital status				
Not married	97	134	1.52 (1.01, 2.29)*	1.32 (0.86, 2.02)α
Married	58	122	1.00	1.00
Number of episodes				
One episode	34	85	1.00	1.00
Two or more episodes	121	171	1.77 (1.12, 2.81)*	1.70 (1.06, 2.74)**
History of aggression				
Yes	141	191	3.43 (1.85, 6.35)*	3.06 (1.63, 5.75)**
No	14	65	1.00	1.00
Education				
No formal education	17	52	2.07 (1.15, 3.73)*	1.73 (0.93, 3.19)α
Educated	138	204	1.00	1.00
Comorbid illness				
Yes	35	27	2.47 (1.43, 4.28)*	2.21 (1.25, 3.90)**
No	120	229	1.00	1.00
Alcohol drinking				
Yes	52	61	1.61 (1.04, 2.51)*	1.28 (0.80, 2.05)α
No	103	195	1.00	1.00

* *P* < 0.05)

** Significantly associated at *P* < 0.05), 1.00 (reference), *α* (not significantly associated)

## Discussion

Patients' lives often change after being diagnosed as mentally ill, and the behavior of patients with bipolar disorder is mostly realted to their illness. Therefore, the response from the environment affects the psychopathology. Inappropriate handling of this behavior and abuse of the patients can occur.

The prevalence of abuse (physical or verbal) is higher in this study compared to studies from New Zealand (27.3%) [29], but lower than the 62.8% reported in the USA [28] and 75% in [30]. Factors such as methodological differences and the clinical and medico-legal factors in each country might be responsible for this inconsistency. There is no universal agreement on how to handle the behavior of patients with mental illness, and the treatment guidelines, laws and ethical issues of each country lead to the discrepancy in the reported abuse among studies.

In the logistic regression analysis, participants who had two or more episodes of bipolar disorder had increased chances of reported abuse—almost two times more than for those who had only one episode [AOR 1.70 95% CI (1.06, 2.74)]. This may be due to the re-occurrence of affective or psychotic symptoms disturbing the family or caregivers, and the counter-response may lead the families or caregivers to mishandle the patients [35]. The episodic nature of the illness itself exposes patients with bipolar disorder to various psychosocial disadvantages such as unemployment, poor social relationships and high costs for their treatment [4]. This psychosocial disadvantage may cause conflicts in the patient's environment that invite abuse. Frequent episodes of illness create various challenges for the patients, including exposing them to abuse [36]. Recurrences of the psychopathology always result in difficult behaviors that distress the caregivers, leading them to be aggressive toward the patients.

Patients’ previous history of aggressive behavior was one contributing factor for reporting abuse, and the odds of reporting abuse were three times larger for those with a reported history of aggressive behavior than for those without [AOR 3.06, 95% CI (1.63, 5.75)]. This had a bidirectional association with aggression and abuse in which most patients who reported abuse had a history of aggression, especially auto-aggression or suicidal attempts [20]. Family- or caregiver-related reports of abuse or mistreatment may be the primary risk factor for aggression in patients with bipolar disorder [37]. This association might be due to the relationship between aggressive behavior and the management of this behavior in the community and health care settings [38].

Comorbid medical and psychiatric illness is common in patients with bipolar disorder, having various impacts on patients' lives. According to this finding, the reported abuse was more than two times greater for patients who had comorbid illness than for those who did not [AOR 2.21, 95% CI (1.25, 3.90)]. This was in line with previous studies finding that illness comorbidities increase the reported abuse [28], and the reported abuse is high among patients with bipolar disorder and comorbid illness [36]. Some disorders can cause behavioral disturbances and may lead patients to develop irritable and aggressive behavior. This may cause different types of responses in the family that lead to patient abuse.

In general, patients who report abuse are not treated in accordance with human rights. The United Nations announced that all persons with a mental illness should be treated with humanity and respect for the inherent dignity of the human person [39]. However, the reported abuse in patients with mental illness has increased in recent studies, including this finding. Therefore, the rights of patients should be considered during their illness.

## Conclusion

Reports of abuse are common in patients with bipolar disorder at psychiatric outpatient clinics in Ethiopia, and this needs public health attention as well as ethical considerations. A history of past aggression, the number of episodes and comorbid illness were found to be significantly associated with abuse.

## Limitations

This was a cross-sectional survey that cannot show the temporal cause-effect association of factors and abuse. This report cannot be generalized for all patients with bipolar disorder in Ethiopia since the study was only carried out in one mental hospital. The tool for assessing abuse is new, and its sensitivity and specificity have been not well reported. All these standardized tools have not been validated in our culture.

## Appendix

### Physical and verbal abuse scale

#### Instruction

- Please encircle “yes” or “no” for each item based on the patient’s experience in relation to his/her family members or caregivers since the onset of the illness.
  1. Physical abuse (any of the following)
    1.1 Hitting
    1.2 Slapping
    1.3 Pushing
    1.4 Throwing any material to harm the patient
  2. Verbal abuse (any of the following)
    2.1 Shouting
    2.2 Insulting
    2.3 Criticizing
    2.4 Undermining patient’s role

#### Scoring

- If the patients answer “one yes” for any of the items, this is considered an experience of reported abuse for each type.
- The internal consistency (Cronbach’s alpha) is 76%.

## Abbreviations

AOR: adjusted odds ratio
CI: confidence interval
WHO: World Health Organization
